# g:Profiler—a web server for functional interpretation of gene lists (2016 update)

**DOI:** 10.1093/nar/gkw199

**Published:** 2016-04-20

**Authors:** Jüri Reimand, Tambet Arak, Priit Adler, Liis Kolberg, Sulev Reisberg, Hedi Peterson, Jaak Vilo

**Affiliations:** 1 Ontario Institute for Cancer Research, 661 University Avenue, Toronto, ON M5G 0A3, Canada; 2 Department of Medical Biophysics, University of Toronto, 101 College Street, Toronto, ON M5G 1L7, Canada; 3 Institute of Computer Science, University of Tartu, Liivi 2, 50409 Tartu, Estonia

## Abstract

Functional enrichment analysis is a key step in interpreting gene lists discovered in diverse high-throughput experiments. g:Profiler studies flat and ranked gene lists and finds statistically significant Gene Ontology terms, pathways and other gene function related terms. Translation of hundreds of gene identifiers is another core feature of g:Profiler. Since its first publication in 2007, our web server has become a popular tool of choice among basic and translational researchers. Timeliness is a major advantage of g:Profiler as genome and pathway information is synchronized with the Ensembl database in quarterly updates. g:Profiler supports 213 species including mammals and other vertebrates, plants, insects and fungi. The 2016 update of g:Profiler introduces several novel features. We have added further functional datasets to interpret gene lists, including transcription factor binding site predictions, Mendelian disease annotations, information about protein expression and complexes and gene mappings of human genetic polymorphisms. Besides the interactive web interface, g:Profiler can be accessed in computational pipelines using our R package, Python interface and BioJS component. g:Profiler is freely available at http://biit.cs.ut.ee/gprofiler/.

## INTRODUCTION

Next-generation sequencing and other high-throughput technologies have revolutionized the characterization of life at molecular resolution. While collection of omics data has become dramatically cheaper and more accessible over the past decades, its interpretation remains a significant challenge. Functional enrichment analysis is a common technique to interpret gene lists. It takes advantage of previous knowledge of gene function and uses a battery of statistical techniques to determine biological processes and pathways characteristic of the genes of interest. Information about biological processes, molecular functions, and cell components and phenotypes is organized into structured vocabularies such as Gene Ontology (GO) (1) and Human Phenotype Ontology (HPO) (2). Databases such as Reactome (3) and KEGG (4) maintain well-curated collections of known molecular pathways. Other functional annotations including protein complexes (5), transcription factor (TF) binding sites (6), microRNA target sites (7) and disease associations (8) can be also used to interpret gene lists. We call all these potential annotations commonly as features or terms that help to interpret the shared properties of the genes in the input lists.

Functional enrichment analysis is a common component of every omics analysis and such resources are in demand in the research community. Many tools of variable qualities are available. While data are frequently updated in some tools such as g:Profiler (9), GOstats (10) and Babelomics (11), many popular tools like DAVID (12) and Bingo (13) have not been updated in years. Tools such as Panther (14), FuncAssociate (15) and GOrilla (16) aim to support the analysis of many species, while others such as WebGestalt (17) focus on the convenient mapping of diverse gene identifiers. Babelomics provides functional enrichment analysis as part of a larger platform (11). While the majority of available tools are web services, functional enrichment analysis can be performed using Java applications (18), R packages (10) and Cytoscape plugins (13). Thus users have many alternatives to interpret their gene lists with functional information. With the ten-year continuous development of g:Profiler, we aim to address the needs of diverse research communities. Our web server provides access to a toolbox of statistical techniques, intuitive interactive analyses, numerous species and a multitude of options.

## g:PROFILER WEB SERVER

The g:Profiler web server (http://biit.cs.ut.ee/gprofiler/) comprises several tools to perform functional enrichment analysis and mine additional information. These tools analyse flat or ranked gene lists for enriched features (g:GOSt; g:Cocoa), convert gene identifiers of different classes (g:Convert), map genes to orthologous genes in related species (g:Orth) and find similarly expressed genes from public microarray datasets (g:Sorter). An additional tool g:SNPense maps human single nucleotide polymorphisms (SNP) to gene names, chromosomal locations and variant consequence terms from Sequence Ontology (19,20).

g:Profiler regularly synchronises with the Ensembl database for gene annotations and identifiers. It supports all species whose genomes are available in Ensembl and Ensembl Genomes (19,21) except for bacterial, archaeal and protist genomes. GO ontologies and some gene annotations are downloaded from the GO website (1). Other functional resources are updated regularly from corresponding databases. The latest versions and dates of each update are documented on our main page.

### g:GOSt—functional enrichment analysis

g:GOSt performs pathway enrichment analysis and is the central tool of the g:Profiler web server. It maps a user-provided gene list to various sources of functional information and determines significantly enriched pathways, processes and other annotations. The GO (1,22) is richest of supported ontologies and is available for many species. We also use molecular pathways from the KEGG (4) and Reactome (3) databases, target sites of miRNAs from the miRBase (7) database, and predicted target sites of TFs using the TRANSFAC resource (6). Information about protein complexes and protein–protein interaction networks from the CORUM database (5) and BioGRID (23) is also used to interpret gene lists. In this update we have included protein expression data from the Human Protein Atlas (HPA) (24). Gene annotations of physiological and disease phenotypes from the HPO (2) and the Online Mendelian Inheritance in Man (OMIM) resource (8) allow users to interpret their gene lists in the context of human health.

g:GOSt supports the majority of gene identifiers used by the basic and biomedical research community. This includes all identifiers that have been linked to genes in the Ensembl database (19), including genes, proteins, transcripts, accession numbers in genome databases, probesets of experimental platforms, etc. For example, g:GOSt recognises 116 types of identifiers of human genes that can be presented as input as a mixed list of genes. This flexible feature allows the user to easily navigate the jungle of numerous omics platforms and gene databases. The gene query can be also presented as a list of chromosomal coordinates. For each chromosomal region we extract all genes that are at least partially located in the given region. Analysis of genes in chromosomal regions is a useful feature for analysing GWAS and epigenomics data.

g:GOSt allows researchers to analyse flat and ranked gene lists. Ranked list analysis is more powerful and is recommended in the majority of cases. In the case of ranked gene lists, first genes in the input list are more important than the following genes (e.g. have a stronger signal in the underlying experiment). g:GOSt then computes a minimum hypergeometric statistic for every term. This technique starts from the top-ranked genes in the list and determines the subset where the enrichment is the strongest. This method provides more resolution to pathway enrichment analysis as it detects both small and highly significant pathways among top-ranked genes as well as broader terms representative of the entire gene list.

We enable and encourage users to provide a custom background for their query when necessary. This statistical technique is essential when the number of genes studied in the specific case is a considerably small subset of all known genes in the genome of the studied gene. For example, certain experimental platforms such as ProtoArrays only cover 1/3 of all human genes and thus the remaining genes are not part of analysis by design. Providing this fraction of genes of as statistical background provides a more accurate estimate of functional enrichment and reduces the bias towards over-interpretation.

g:GOSt applies the widely applied hypergeometric distribution to estimate the significance of enriched pathways and processes in gene lists. Each default analysis of human gene lists considers more than 30 000 gene sets corresponding to a large variety of features. Thus a multiple testing correction is required to reduce false positive findings. With the first release of g:Profiler in 2007 we introduced a ontology-focused multiple testing correction method g:SCS (9). We showed that the most common multiple testing correction methods incorrectly estimate the expected number of false positive results in the enrichment analysis: the Benjamini–Hochberg False Discovery Rate tends to find more false positives while the Bonferroni correction is overly conservative. The g:SCS method is used by default in g:Profiler and users can choose to use the other two correction methods.

g:GOSt allows users to filter resources used to interpret gene lists. For example, one may choose to only use biological processes of GO and Reactome pathways and filter out other databases and ontologies. Similarly, one may focus on relatively small biological processes (more than five genes and less than five hundred) and discard other gene sets prior to analysis. Such filtering speeds up calculations, improves statistical power and reduces the effect of multiple testing, as well as provides easier interpretation. We recommend users to consider data resources beforehand and select the most interesting ones for their particular analysis.

The main output of g:GOSt comprises a visual matrix of functional annotations of genes. Each gene in the input list is highlighted with a coloured square if it belongs to the respective enriched term. Colours represent different evidence codes for GO as well as gene annotations to other functional resources. Several metrics are also reported for each of the enriched results, including the size of the gene set in question, overlap with the input gene list and the statistical significance (*P*-value). Individual results are grouped by their hierarchy relative to other results, or alternatively ordered by *P*-value. Additional details about the query are given below the visualization, along with statistical background sizes, the input gene list and involved protein interaction networks. The results are either provided in graphical format (PNG, PDF), text file or Excel spreadsheet.

We also look for enriched modules in BioGRID protein–protein interaction (PPI) network (23). Input genes that have at least one common interaction partner in the PPI network are visualized with all their partners. We use the Cytoscape.js JavaScript library (25) to visualize these networks and provide all interaction data as text.

g:GOSt results can be easily integrated with the Enrichment Map (26) method that provides network visualization of functional enrichment analysis. Enrichment Map is a useful method for simplifying complex results with many redundant processes and gene functions. g:GOSt provides a special output format (generic enrichment map) that can be directly uploaded into Cytoscape for visual network analysis.

### g:Cocoa—simultaneous enrichment analysis of multiple gene lists

g:Cocoa provides means to analyse several gene lists at the same time and compare their characteristic enriched terms. This is useful in scenarios where an experimental design involves many comparisons of samples or individuals, or when one wants to compare directly different clusters of genes arising from the analysis. Each gene list is analysed for functional enrichments similarly to g:GOSt and resulting terms are aligned vertically into a matrix highlighting strongest findings for every gene list.

### g:Convert—automatic conversion of gene identifiers

g:Convert provides a convenient service to translate identifiers (IDs) of genes, proteins, microarray probesets and many other types of namespaces. The seamless translation process works on a mixed set of diverse identifiers and maps these through Ensembl gene identifiers (ENSG) as reference. In cases of multiple identifiers, all relevant combinations are highlighted. At least 13 types of IDs are supported for all of the 213 species available in g:Profiler, and at least 40 types of IDs for more than 50 species.

### g:Orth—mapping related genes across species

g:Orth allows the user to map a list of genes of interest to homologous genes in another related organism. Many experiments are conducted in model organisms and knowledge from such experiments is transferred to other organisms to compare or complement previous findings. g:Orth uses g:Convert to map gene IDs to Ensembl ENSG identifiers. Further mapping to orthologous genes in other organisms is also based on Ensembl data (19,21). We provide cross-references between all organisms in g:Profiler. Queries are limited to tiers according to classes of species (animals, plants, fungi).

### g:Sorter—finding similar genes in transcriptomics data

g:Sorter is a tool for finding lists of co-expressed genes from public transcriptomics datasets. Thousands of microarray and RNA-seq experiments have been conducted in the past decades. The majority of published studies have been accumulated in databases like ArrayExpress (27) and Gene Expression Omnibus (28). We have downloaded 7878 datasets for 18 species from ArrayExpress and provide gene co-expression similarity searches using six most common statistical methods. The datasets can be searched rapidly with keywords. The input of g:Sorter is a single gene and a dataset of interest and the result is a sorted list of genes that are similarly expressed with the gene of interest. These lists can be integrated into functional enrichment analysis. For comprehensive global gene expression similarity queries, as well as support for more species and platforms we suggest to use Multi Experiment Matrix (MEM) tool (29).

### gProfileR package in R for automated analyses

The g:Profiler web server can be accessed in GNU R using the dedicated R package gProfileR available in CRAN. The R is a core asset of the bioinformatics community with hundreds of resources and analysis packages available. We provide the R package to enable integration of our tools to diverse automated pipelines. The package accesses our web server via the internet and covers the functionality of g:GOSt, g:Cocoa, g:Convert and g:Orth.

## NEW DEVELOPMENTS IN G:PROFILER IN 2016

Since our previous publication in 2011 (30), we have added several new resources for interpreting gene lists and implemented new technologies. With our data update and archiving policy, we aim to maximize reproducibility and timeliness of research.

### Mapping of ambiguous gene identifiers

Gene identifier mapping is a complex problem as the community continuously replaces earlier identifiers by newer ones and multiple aliases are the rule rather than an exception. This creates ambiguities in gene list interpretation and may cause genes to be excluded. To remedy this situation, we now provide semi-manual mapping of gene identifiers in addition to our automated annotation pipeline. We determine input genes that cannot be mapped to single ENSG identifiers and provide these to the user as an optional form where correct identifiers can be selected manually or excluded from the analysis. This approach guarantees that important genes are always included in the enrichment analysis.

### Transcription factor binding site predictions with TRANSFAC

**Figure 1.**
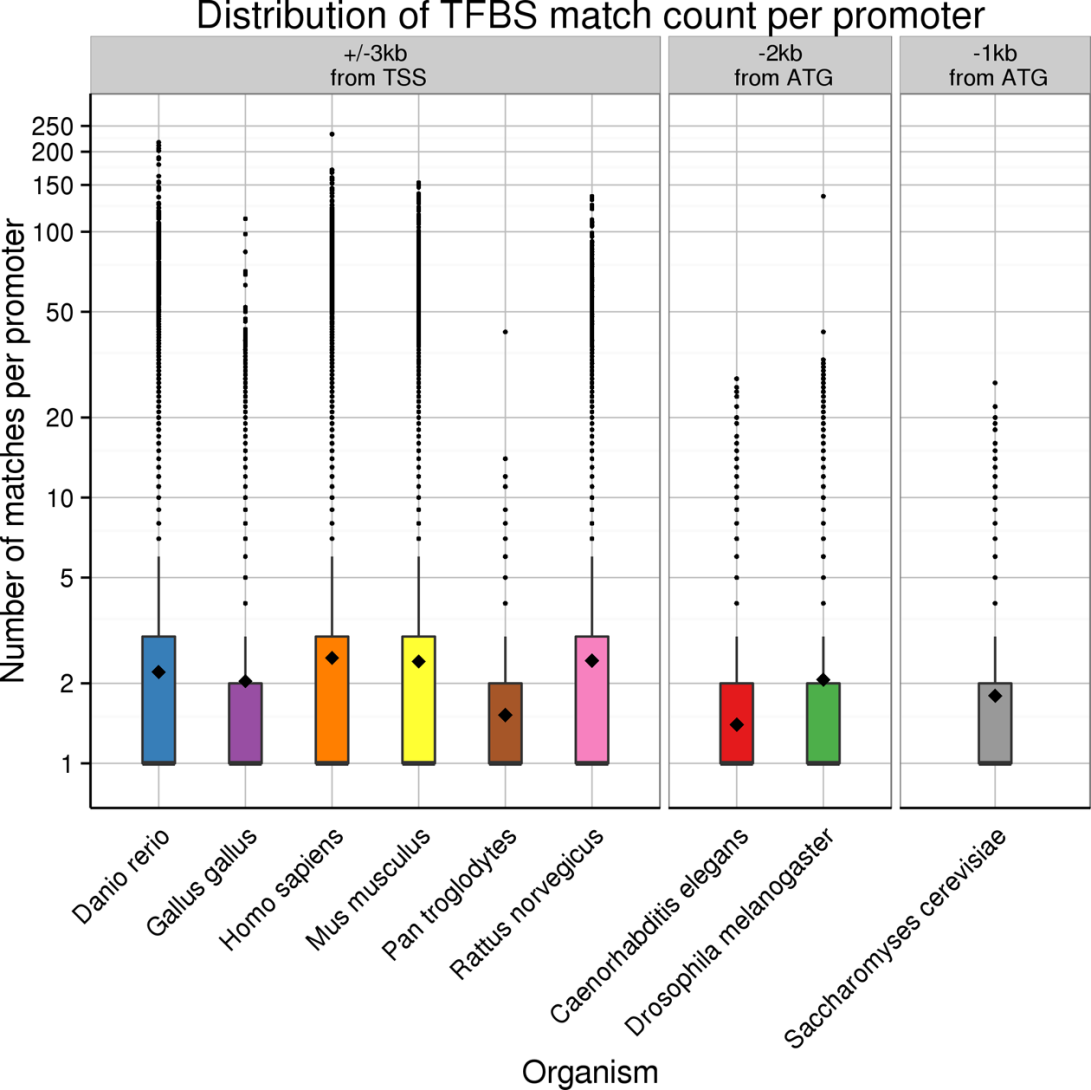
We predicted regulatory motifs from the TRANSFAC database for nine species shown on the x-axis. We used 6 kb promoter regions (±3 kb) upstream the transcription start sites for vertebrates, 2 kb promoters for fly and worm and 1000 bp promoters for yeast. TFBS matches per promoter are given with boxplots on the y-axis where the mean number of sites per promoter per TF is depicted with a black diamond.

TF binding in regulatory DNA determines regulation of gene expression. Thus information about TF binding sites (TFBS) can be used to interpret gene lists and enrichment of TFBS in gene promoters may indicate common regulation and biological function. We have updated our binding site predictions in gene promoters by systematically mapping TF binding motifs to regulatory DNA in multiple species including human, mouse, chicken, fly and yeast. The promoter sizes depend on the species and are depicted in Figure 1.

We have updated TFBS data in g:Profiler and changed our definitions of potential regulation events. We used regulatory motifs in the TRANSFAC database version 2015.3 to make computational predictions of binding sites in gene promoters. We use the TRANSFAC internal threshold for limiting false positive matches (minFP) of TFBS. On average, each promoter has between 1.4 and 2.5 TFBS on average for included species. Thus we introduce a two-step hierarchy of terms where the upper more lenient category covers all the genes that have at least one match of the given TFBS in their promoter, while the second more stringent category covers promoters where the motif needs to be present at least twice. The second category with stronger binding sites suggests a stronger regulatory relation.

### Enriched protein expression patterns from HPA

The HPA is a compendium of protein expression in 44 normal human tissues measured by immunohistochemistry (24). Protein expression levels are categorized into four groups (not detected, low, medium and high expression) with two evidence terms of presence (uncertain, supportive). To allow interpretation of gene lists using this information, we have mapped gene sets corresponding to protein expression signatures into a hierarchy of terms that reflects their tissue-specific level of expression. The most stringent terms include only highly expressed proteins per tissue, while lenient terms include highly as well as lowly expressed proteins. The HPA resource provides 713 tissue-specific groups of genes corresponding to 15 000 genes.

### Enrichment of Mendelian disorders

OMIM is a collection of human genes and their relationships with Mendelian disorders and other genetic phenotypes (8). Although the majority of OMIM descriptions are included already into g:Profiler via the HPO, we have also directly added more than 4500 OMIM annotations to 3500 genes and provide methods to search for over-represented disorders. Disorders have been organized hierarchically to parental terms using information on genetic heterogeneity in OMIM data records.

### Genomic and functional data for 213 species

**Figure 2.**
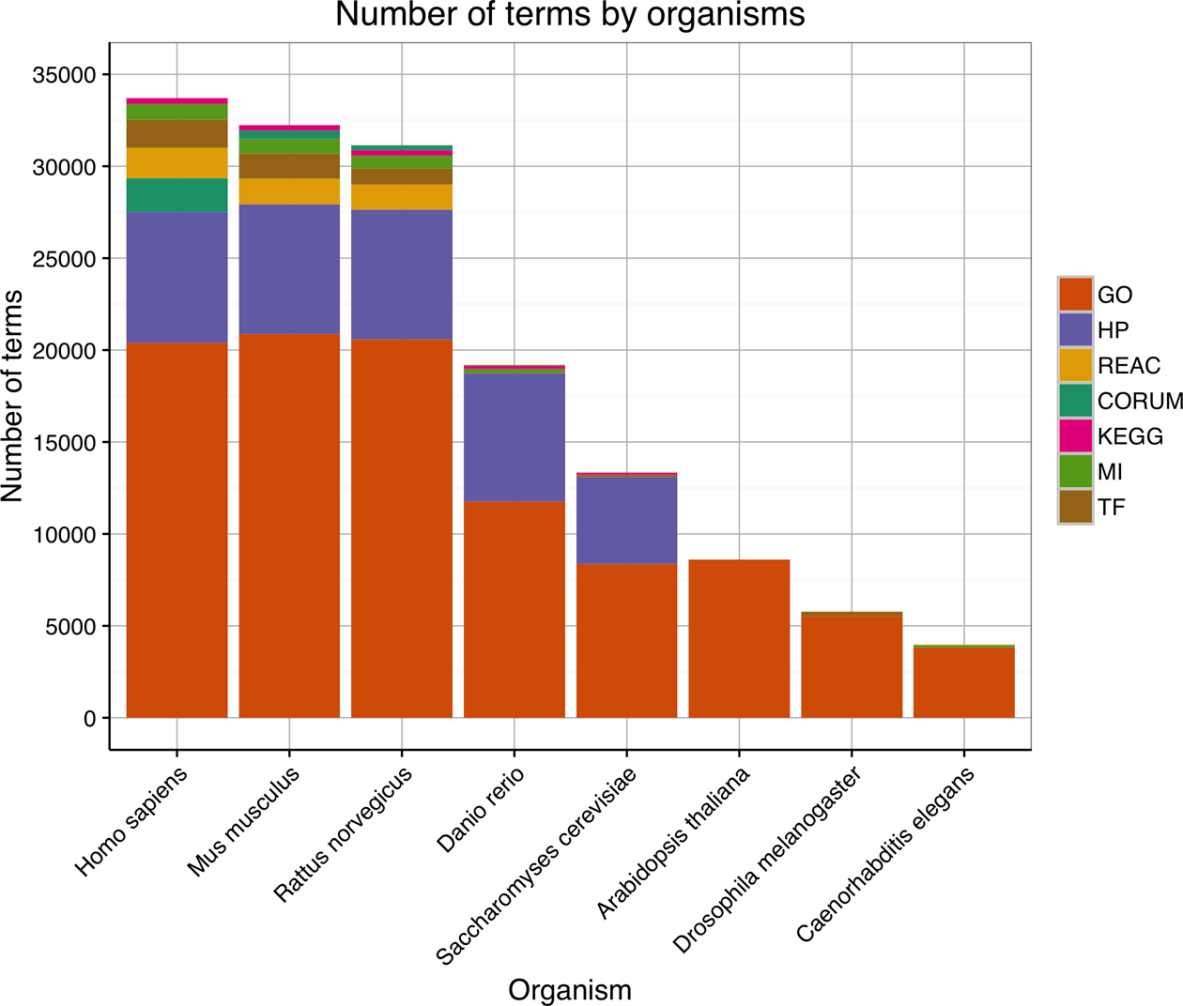
Total number of annotations terms per eight most common organisms in g:GOSt. Each colour represents different functional resource and the largest one (GO) is depicted with orange. Supplementary Figure S1 shows a similar overview of all 213 organisms.

The 2016 version of g:Profiler supports the analysis of data from 213 different organisms from Ensembl (19) and Ensembl Genomes (21). g:Profiler covers 67 vertebrate, 38 plant and 52 fungi species among others, nearly doubling from 126 in our previous update in 2011 (30) (Figure 2). This makes g:Profiler the most species-rich functional enrichment analysis tool serving different research communities in life sciences.

### g:SNPense—SNP identifier mapping

With the rapid growth of whole genome sequencing technology, researchers are uncovering extensive genetic variation and large collections of known SNP are available for human and other species. In order to easily map SNP identifiers (e.g. rs4244285) to gene names, chromosomal coordinates and retrieve their functional consequences we now provide a new service called g:SNPense. Information about genome variants is retrieved from dbSNP (31) and mapped to NCBI Genes (32). Potential functional consequences of the variants are retrieved from Ensembl Variation data (19) and grouped into 35 Sequence Ontology terms of various severity (20). g:SNPense is a potential entry point to g:GOSt and its functional annotation pipeline and enrichment analysis.

### Programmable access to g:Profiler

The research community increasingly requires automatic and programmable access to web tools as basic and biomedical science is becoming increasingly data intensive. In addition to the CRAN-supported R package gProfileR that we have been providing for years, we are now expanding the programmable access capabilities to new technologies. We provide an application program interface (API) with Python that can be included into user-friendly bioinformatics analysis software such as Chipster (33) and Galaxy (34), allowing users to set up their own custom analytical pipelines for large-scale analysis. We already provide the g:Profiler utility as part of the Galaxy ToolShed (35).

### BioJS component for visualizing g:Profiler results as word clouds

**Figure 3.**
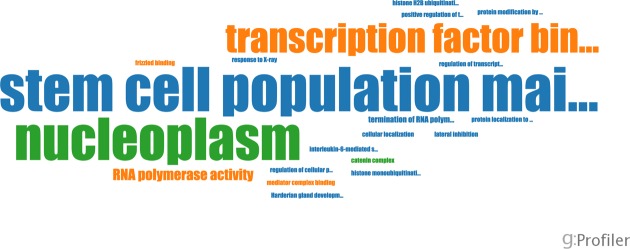
BioJS output from the MEM tool depicting processes and functions related to embryonic stem cells.

BioJS is an open source bioinformatics project comprising a library of JavaScript components for visualising biological data in web applications (36). We have developed a BioJS component for g:Profiler (biojs-vis-gprofiler) that performs g:GOSt analysis and represents the most significant keywords as word clouds. Clicking on keywords reveals associated biological processes and enrichment statistics. This simplified solution can be used in web applications and pipelines such as our MEM tool (29) where a comprehensive visual representation is not required. Tag clouds provide an easily interpretable visualization of most common terms highlighted with different colours and font sizes (Figure 3).

### Data maintenance policy for reproducibility

Since the publication in 2011 (30) all 13 previous releases of g:Profiler have been saved on our web server and are accessible on the web site through the dedicated link to Archive. This allows users to continue or verify their analysis on the same set of data that was available at the time of the original analysis. With this policy, we aim to increase transparency and integrity of bioinformatics data analysis.

**Figure 4.**
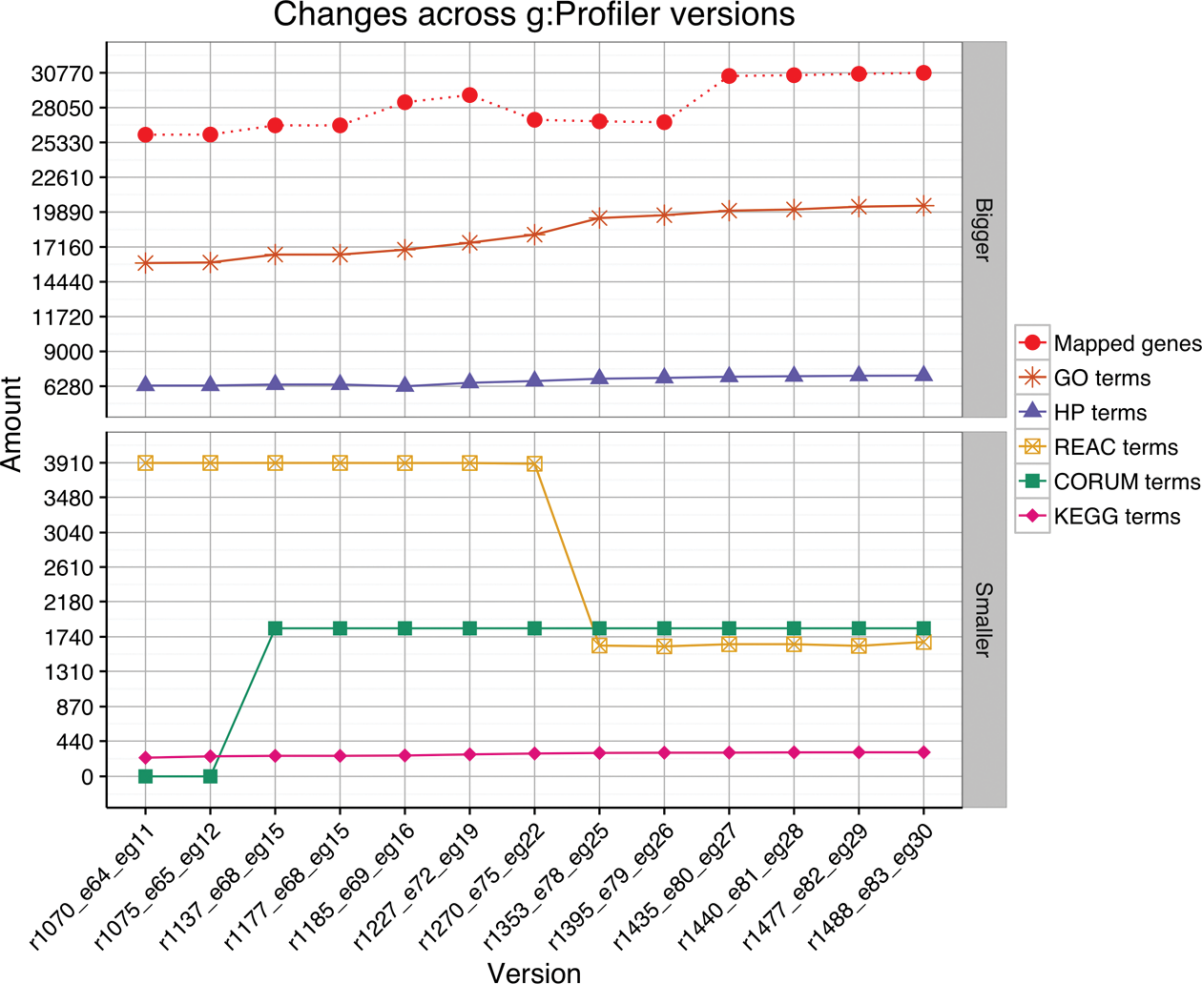
Since 2011 the underlying human datasets in g:Profiler have been steadily growing. The majority of the functional resources have grown as depicted over the 13 latest g:Profiler versions. The number of mapped human genes has also grown by 20% from 26 000 to 31 000. As an exception, the Reactome dataset has decreased as we filtered out gene sets corresponding to reactions starting from g:Profiler version R1353.

Since December 2014, g:Profiler has been updated on a quarterly basis following each Ensembl release (Figure 4). This assures that central gene identifier indexes and GO annotations are never older than 6 months. This renders g:Profiler one of the most up to date functional enrichment tool available today. While resources such as Reactome, KEGG, HPO, OMIM and others follow their own release schedules and add new genes to their databases, we check for updates in these resources when we conduct the main cycles with Ensembl. Thus some more static resources are updated less frequently (e.g. regulatory motifs of microRNAs and TFs). In addition to the stable g:Profiler version, we also provide public access to a development server called g:Profiler Beta for power users who benefit from the latest developments and newest data sources.

## DISCUSSION

With the continuous development of g:Profiler, combining further resources and releasing programmable access points (web, R, Python API, BioJS), we aim to provide a state-of-the-art functional profiling and identifier mapping service. Our tool combines up-to-date functional and genomic data with sophisticated algorithms and serves the community through an intuitive and freely accessible website with efficient visualization techniques.

With this update we introduce more datasets for functional interpretation of gene lists. These include physiological and disease-related gene sets from OMIM, tissue-specific protein resources from the HPA and a new release of gene regulatory predictions using the TRANSFAC resource. We also have a new SNP identifier translation service g:SNPense. Since the last publication in 2011 we have more than doubled the number of supported species to 213 species. This is the largest number of species supported by any of the publicly available functional enrichment tools.

In this update we focus on programmable ways to access our service. We have developed the Python API to g:Profiler that provides means to include our analysis to bioinformatics pipelines using solutions like Galaxy or Chipster (33,34). We have increased the number of output formats on our web portal for easier downstream analysis. We have also developed the g:Profiler BioJS JavaScript component that can be incorporated into independent websites.

We consider data timeliness our highest priority. Novel annotation terms and findings about gene functions appear daily and therefore it is important to promptly consider this information for functional annotation. Annotating gene lists with data from five years ago, like when using DAVID (12), provides different conclusions than current data would. This is especially important in fields of research where technological improvements have only recently allowed high-throughput analysis (e.g. single cell analysis, embryonic stem cells, precision medicine).

The g:Profiler service has recently proven to be useful to a broad user community studying anything from insects to wolves and plants (37–39). The most frequent use cases of g:Profiler probably relate to cancer genomics (40–44), stem cell research (45,46) and ageing (47,48). g:Profiler is a recommended tool for interpreting cancer genomes with pathway information (49).

Several bioinformatics tools have incorporated the functionality of g:Profiler through dedicated APIs. For example, the global gene expression similarity analysis tool MEM (29) uses g:Convert for identifier mapping and the BioJS library for summarising enrichment results. Our online multivariate data clustering and visualization tool ClustVis (50) uses data and name mapping services from g:Profiler. A similar approach to GO-based word clouds is also used in the R package GOsummaries (51) that combines enrichment analysis of gene lists from g:GOSt with principal component analysis of gene expression data.

Future developments of g:Profiler will focus on research of precision medicine and also on supporting as many species as possible. Advances in whole genome sequencing technology create requirements for novel tools that analyse genome variation for functional enrichments, their relation to drugs and diseases, protein domains or phosphorylation sites (52,53). As the enrichment analysis and identifier mapping services are highly needed for many species, our goal is to support the research of common and uncommon model organisms.

## Supplementary Material

SUPPLEMENTARY DATA
