# Supplementary material for: g:Profiler—a web server for functional interpretation of gene lists (2016 update)
Source: Nucleic Acids Res. 2016 Apr 20;44(Web Server issue):W83–9. doi: 10.1093/nar/gkw199 (PMC4987867; doi:10.1093/nar/gkw199)
Supplement: SUPPLEMENTARY DATA [file supp_44_W1_W83__index.html]

g:Profiler—a web server for functional interpretation of gene lists (2016 update) — g:Profiler—a web server for functional interpretation of gene lists (2016 update) — g:Profiler—a web server for functional interpretation of gene lists (2016 update) — SUPPLEMENTARY DATA 

# g:Profiler—a web server for functional interpretation of gene lists (2016 update)

## SUPPLEMENTARY DATA

- SUPPLEMENTARY DATA
