# Supplementary figures and images for: g:Profiler—a web server for functional interpretation of gene lists (2016 update)
Source: Nucleic Acids Res. 2016 Apr 20;44(Web Server issue):W83–9. doi: 10.1093/nar/gkw199 (PMC4987867; doi:10.1093/nar/gkw199)

Number of terms by organisms

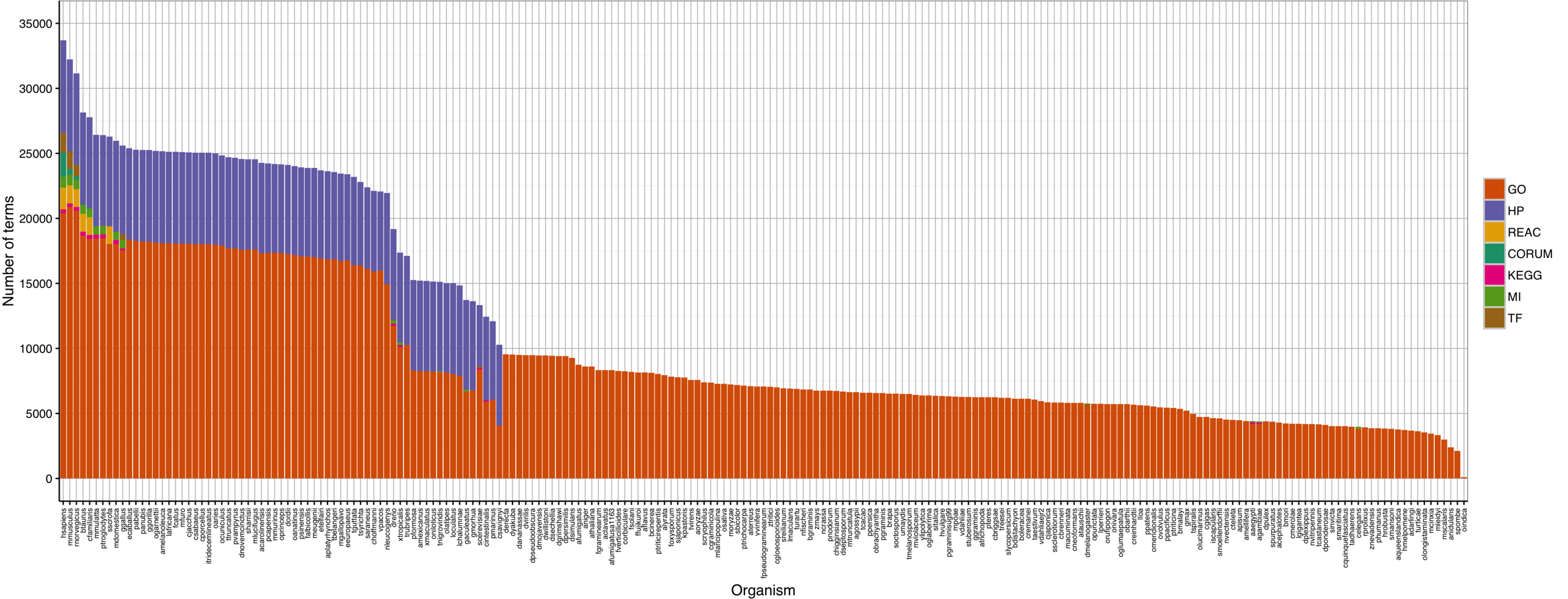

Supplement: SUPPLEMENTARY DATA [file supp_gkw199_nar-00301-web-b-2016-File002.pdf]
